# Complete mitochondrial genome of *Actias dubernardi* (Lepidoptera: Saturniidae)

**DOI:** 10.1080/23802359.2021.1875929

**Published:** 2021-02-17

**Authors:** Fang Zhao, Tianjuan Su, Bo He, Kai Jiang, Chuanxin Zuo, Gonghua Lin, Zuhao Huang

**Affiliations:** a School of Life Sciences, Jinggangshan University, Ji’an, China; b Administrative Bureau of Jinggangshan National Nature Reserve, Jinggangshan, China

**Keywords:** Saturniidae, *Actias dubernardi*, mitogenome, phylogeny

## Abstract

The complete mitochondrial genome of *Actias dubernardi* (Lepidoptera: Saturniidae) is 15,270 bp in length, containing 13 protein-coding genes, 22 transfer RNAs, 2 ribosomal RNAs, and a putative control region. All of the protein-coding genes (PCGs) use the standard start codon ATN, except for cox1 which starts with CGA. The Bayesian phylogenetic analysis was performed using a dataset matrix containing 13 PCGs concatenated from the mitogenomes of 14 Saturniidae species. The monophyly of the five *Actias* species was highly supported and *Antheraea* was inferred as the sister group of *Actias*.

Saturniidae (Lepidoptera) is a widely distributed family of moths including some of the largest known insects. A total of 3454 species belonging to 180 genera have been identified in Saturniidae (Kitching et al. 2018). Many Saturniidae species are important economic insects. For example, at least 11 African Saturniidae species such as *Gonimbrasia belina* and *Gynanisa maja* are consumed at the caterpillar stage (Langley et al. 2020), while some Asian species such as *Antherea pernyi* and *Philosamia cynthia ricini* are reared for silk production (Liu et al. 2010). Many Saturniidae species such as *Actias spp.*, *Loepa spp.*, and *Saturnia spp.*, which are strikingly beautiful, have significant economic importance that is associated with ornamental value (Wu 2017). *Actias dubernardi* (Oberthur 1897), the Chinese moon moth, is a Saturniidae species that originates from southern China. With extremely long cercus and gorgeous brilliant colors, *A. dubernardi* is viewed as one of the most beautiful moths in the world. Unfortunately, however, very little was known about the biology (but see Naumann 2006) of this species. Here, we present the mitochondrial genomes of *A. dubernardi* and tested its phylogenetic relationship with other Saturniidae species whose mitogenomes were available in the GenBank.

The specimen was collected from Jiangxi Province, China (N26.51°, E114.10°) in May 2020, and was preserved at Entomological Specimen Room of Jinggangshan University (accession number: 202005-Lep027). Total genomic DNA was extracted from a single specimen and was sequenced by Illumina HiSeq2000, with pairwise reads of 150 bp. The complete mitogenome was assembled by the GetOrganelle v1.6.4 program (Jin et al. 2020). The mitogenome structure was annotated using the MITOS2 webserver (Bernt et al. 2013) and was checked according to two closely related species (*Actias selene*, NC_018133.1; *Actias luna*, NC_045899.1). Furthermore, we checked the coding sequences (CDSs) by nucleotide BLAST and the CDS feature display.

The complete mitogenome of *A. dubernardi* is 15,270 bp in size, composed 37 genes as in most insect mitogenomes (Cameron 2014), including 13 protein-coding genes (PCGs), 2 ribosomal RNAs (*16S* and *12S*), 22 transfer RNAs (tRNAs), along with the noncoding control region, termed in insects as A + T rich region (GenBank accession no. MW133617). The gene arrangement of the *A. dubernardi* was identical to the majority of the Lepidoptera (Wan et al. 2013), with the order *trnM*/*trnI*/*trnQ* between the A + T rich region and *nad2*. The overall base composition is 38.7% A, 39.5% T, 13.5% C, and 8.3% G. The nucleotide composition of the *A. dubernardi* mitogenome is biased toward A + T (78.2%). All of the PCGs use the standard start codon ATN, except for cox1 which starts with CGA. The *16S* is located between *trnL1* and *trnV*, with a length of 1371 bp. The *12S* is located between *trnV* and the control region, with a length of 779 bp. The control region is 330 bp in length, and is located between *12S* and *trnM*.

Phylogenetic analysis was performed on concatenated nucleotide sequences of the 13 PCGs of five *Actias* species, along with nine Saturniidae species as an outgroup (Langley et al. 2020). Sequences of the 14 species of each PCG were aligned using CLUSTAL X (Thompson et al. 1997) with the default settings and refined manually. The alignment of each PCG was deliberately trimmed to equal length before concatenating. Phylogenetic inference was performed using MrBayes 3.2.7 (Ronquist et al. 2012) with 10,000,000 generations, which are sufficient to meet the 0.01 criteria of standard deviation of split frequencies. The best-fit nucleotide substitution models (GTR + I + G) were determined by AIC implemented in jModelTest 2 (Darriba et al. 2012). As expected, the monophyly of *Actias* species was supported with 100% bootstrapping rates. *A. dubernardi* was inferred as the sister group of a subclade including *A. luna*, *A. selene*, and *A. artemis*, while *A. maenas* had the basal status in the genus (Figure 1). In addition, *Antheraea* was inferred as the sister group of *Actias*, consistent with that previously described (Langley et al. 2020).

**Figure 1.**
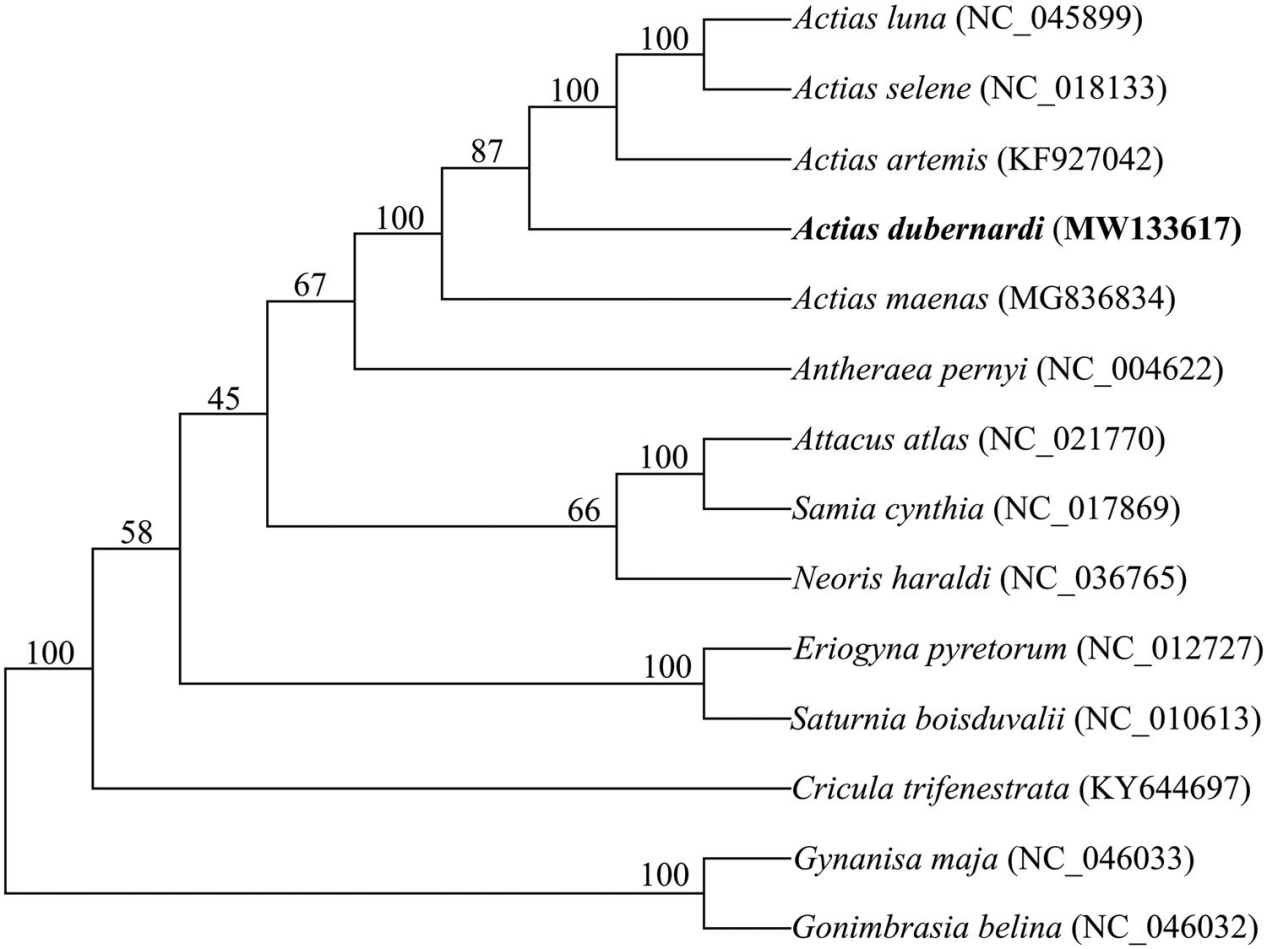
Phylogenetic analyses of mitochondrial genomes of *Actias dubernardi* and 13 other Saturniidae species. The focal mitochondrial genomes in this study are in bold. Numbers beside each node represent percentages of Bayesian bootstrap values. Species names are followed by the GenBank accession numbers of their mitochondrial genomes.

## Data Availability

The mitogenome sequence data that support the findings of this study are openly available in GenBank of NCBI at (https://www.ncbi.nlm.nih.gov/) under the accession no. MW133617. The associated BioProject, SRA, and Bio-Sample numbers are PRJNA687294, SRR13329533, and SAMN17141016, respectively.
